# African American race does not confer an increased risk of clinical events in patients with primary sclerosing cholangitis

**DOI:** 10.1097/HC9.0000000000000366

**Published:** 2024-01-29

**Authors:** Maryam Yazdanfar, Joseph Zepeda, Richard Dean, Jialin Wu, Cynthia Levy, David Goldberg, Craig Lammert, Stacey Prenner, K. Rajender Reddy, Daniel Pratt, Lisa Forman, David N. Assis, Ellina Lytvyak, Aldo J. Montano-Loza, Stuart C. Gordon, Elizabeth J. Carey, Joseph Ahn, Barry Schlansky, Joshua Korzenik, Raffi Karagozian, Bilal Hameed, Shaun Chandna, Lei Yu, Christopher L. Bowlus

**Affiliations:** 1 Division of Gastroenterology and Hepatology, University of California Davis, Sacramento, California, USA; 2 Division of Gastroenterology and Hepatology, Schiff Center for Liver Disease, University of Miami, Miami, Florida, USA; 3 Indiana University, Indianapolis, Indiana, USA; 4 Division of Gastroenterology and Hepatology, University of Pennsylvania, Philadelphia, Pennsylvania, USA; 5 University of Colorado, Denver, Colorado, USA; 6 Yale University, Hew Haven, Connecticut, USA; 7 Division of Preventive Medicine, University of Alberta, Edmonton, Alberta, Canada; 8 Division of Gastroenterology and Liver Unit, University of Alberta, Edmonton, Alberta, Canada; 9 Henry Ford Health and Wayne State University School of Medicine, Detroit, Michigan, USA; 10 Mayo Clinic Arizona, Scottsdale, Arizona, USA; 11 Oregon Health Sciences University, Portland, Oregon, USA; 12 Kaiser Permanente Northwest, Portland, Oregon, USA; 13 Brigham & Women’s Health, Boston, Massachusetts, USA; 14 Tufts Medical, Boston, Massachusetts, USA; 15 UC San Francisco, San Francisco, California, USA; 16 University of Utah, Salt Lake City, Utah, USA; 17 University of Washington, Seattle, Washington, USA

## Abstract

**Background:**

The natural history of primary sclerosing cholangitis (PSC) among African Americans (AA) is not well understood.

**Methods:**

Transplant-free survival and hepatic decompensation–free survival were assessed using a retrospective research registry from 16 centers throughout North America. Patients with PSC alive without liver transplantation after 2008 were included. Diagnostic delay was defined from the first abnormal liver test to the first abnormal cholangiogram/liver biopsy. Socioeconomic status was imputed by the Zip code.

**Results:**

Among 850 patients, 661 (77.8%) were non-Hispanic Whites (NHWs), and 85 (10.0%) were AA. There were no significant differences by race in age at diagnosis, sex, or PSC type. Inflammatory bowel disease was more common in NHWs (75.8% vs. 51.8% *p*=0.0001). The baseline (median, IQR) Amsterdam-Oxford Model score was lower in NHWs (14.3, 13.4–15.2 vs. 15.1, 14.1–15.7, *p*=0.002), but Mayo risk score (0.03, −0.8 to 1.1 vs. 0.02, −0.7 to 1.0, *p*=0.83), Model for End-stage Liver Disease (5.9, 2.8–10.7 vs. 6.4, 2.6–10.4, *p*=0.95), and cirrhosis (27.4% vs. 27.1%, *p*=0.95) did not differ. Race was not associated with hepatic decompensation, and after adjusting for clinical variables, neither race nor socioeconomic status was associated with transplant-free survival. Variables independently associated with death/liver transplant (HR, 95% CI) included age at diagnosis (1.04, 1.02–1.06, *p*<0.0001), total bilirubin (1.06, 1.04–1.08, *p*<0.0001), and albumin (0.44, 0.33–0.61, *p*<0.0001). AA race did not affect the performance of prognostic models.

**Conclusions:**

AA patients with PSC have a lower rate of inflammatory bowel disease but similar progression to hepatic decompensation and liver transplant/death compared to NHWs.

## INTRODUCTION

Primary sclerosing cholangitis (PSC) is a fibrotic disease of the bile ducts that is frequently associated with inflammatory bowel disease (IBD) and can lead to cirrhosis, hepatic decompensation (HD), and hepatobiliary malignancies.^1^ Despite the significant efforts and numerous clinical trials, there are no approved medical therapies, and liver transplantation remains the only effective treatment in patients with HD or recurrent episodes of cholangitis.^2^,^3^,^4^,^5^

Studies from referral centers report median postdiagnosis liver transplant-free survival (TFS) of 15 years,^6^ but estimates upward of 21 years have been reported by more recent population-based studies of patients not followed at liver transplant centers.^7^,^8^,^9^ In the largest natural history study to date, older age at diagnosis, male sex, and large-duct PSC were associated with lower TFS, while Crohn disease was associated with better TFS.^6^ However, the majority of epidemiologic and natural history studies of PSC are derived from Scandinavian and Northern European countries, with a paucity of representative data from North American or other non-European populations.

Several models of TFS and HD-free survival have been developed for patients with PSC.^10^,^11^,^12^,^13^,^14^ The Mayo Risk Score (MRS) predicts short-term mortality and has traditionally been the most widely used.^13^ More recent models have been developed to predict longer-term TFS^12^,^14^ and HD^11^ as well as outcomes in children with PSC.^10^ Although several of these models have been validated in additional cohorts, their performance in racially and ethnically diverse populations has not been confirmed.

We previously demonstrated that PSC occurs in African Americans (AA) with a similar frequency as non-Hispanic Whites (NHWs).^15^ In addition, AA patients with PSC listed for liver transplantation were younger, more frequently female, had higher Model for End-stage Liver Disease (MELD) scores, and had a lower frequency of IBD compared to NHWs, suggesting differences in the clinical features and natural history of PSC among AAs.^16^ Similarly, an analysis of patients with PSC with IBD in the United Kingdom found that non-Whites were diagnosed at a younger age, and likewise, African Caribbean heritage was associated with a greater risk of transplantation or PSC-related death.^9^ Using data from a large, multicenter, racially/ethnically diverse sample of patients with PSC from North America that was established by the Consortium for Autoimmune Liver Disease (CALiD), we sought to compare the natural history of PSC between AAs and NHWs and to determine if prognostic models of PSC outcomes perform similarly for AA with PSC.

## METHODS

### Study population

CALiD has developed a contemporary retrospective/prospective research registry involving 16 centers throughout North America (Supplemental Table S1, http://links.lww.com/HC9/A760). The sample includes all patients with PSC alive in January 2008 or later, without a liver transplant prior to January 2008, and with at least 1 encounter after January 2008 at each center. Diagnostic criteria for PSC were consistent with current recommendations.^1^ Patients with cholangiographic features typical of PSC were classified as large-duct PSC; those with normal cholangiograms but liver biopsies and other clinical features consistent with PSC were classified as small-duct PSC. PSC-AIH overlap was diagnosed based on the site investigator’s clinical opinion. Relevant patient data collected from January 2008 through February 2022 included demographics, laboratory data at baseline and annually, pathology, imaging, endoscopy, surgery, and clinical events. Living patients who have not received a liver transplant are entered into the prospective study arm; their data are updated annually. Each site enters data into a centralized REDCap database. For the current analysis, only retrospective data were included.

All research was approved by the institutional review board of each participating site and was conducted in accordance with the Declaration of Helsinki and Istanbul guidelines of good practice. All patients enrolled in the prospective arm provide informed consent. For patients enrolled only in the retrospective arm, only deidentified data were collected, and the requirement for informed consent was waived at each center.

### Data interpretation and statistical analysis

The primary end point was liver transplantation or death, whichever was earliest. Secondary end points included HD (defined as the composite end point of any of the following): ascites, variceal bleeding, or HE. Patients were followed from the time of PSC diagnosis (defined as the date of the first abnormal cholangiogram or liver biopsy). When available, the earliest known abnormal liver biochemistry was also recorded. MRS, Amsterdam-Oxford Model (AOM), and MELD scores were calculated as described.^13^,^14^ Diagnostic delay was calculated as the time from the first abnormal liver biochemistry to the first abnormal cholangiogram or liver biopsy; data were log-transformed to account for positive skewness. Statewide median income by ZIP code was used as a surrogate for socioeconomic status (SES) and was based on the 2015 American Community Survey.^17^ Cirrhosis was defined by cirrhosis on liver biopsy, cirrhotic morphology on imaging, transient elastography >14.4 kPa, magnetic resonance elastography >4.93 kPa, or aspartate aminotransferase (AST) to Platelet Ratio Index >2 based on prior studies establishing these cutoffs (Supplemental Table S2, http://links.lww.com/HC9/A760).^18^,^19^

The Mann-Whitney *U* test or Kruskal-Wallis test was applied as appropriate for continuous variables. Data are presented as median (IQR) except for diagnostic delay, which is presented as median (range) and was assessed after log transformation. The Kaplan-Meier method was applied to explore associations between TFS and race and TFS and tertiles of SES; significance was tested by the log-rank test. Time-to-event analysis was performed using Cox proportional hazards regression analyses. Variables with a prognostic significance <0.1 were entered into a multivariable model to identify those independently related to TFS. Harrell c-index was used to compare MRS, AOM, and MELD in the total cohort. All statistical analyses were performed with SAS (version 9.4, SAS Institute, Cary, NC) and Stata SE (version 17, College Station, TX). *p*-values <0.05 were considered significant.

## RESULTS

### Cohort characteristics

A total of 850 patients were included in the data set; 661 (77.8%) were NHWs, 85 (10.0%) were AA, 50 (5.9%) were Hispanic Whites (HW), and 54 (6.4%) were other/unknown (Table 1). There were no significant differences between NHWs, AAs, and HWs for age at diagnosis based on first abnormal liver biochemistry or age at first abnormal cholangiogram or liver histology. Sex and PSC types were not different. As previously reported, IBD was less common in AA patients compared to NHWs (51.8% vs. 75.8% *p*=0.0001), and the rate of colonoscopy was not significantly different between these groups (76.5% vs. 79.4%, *p*=0.34) supporting a true difference in IBD prevalence. In addition, the frequency of prevalent IBD at PSC diagnosis, defined as an IBD diagnosis more than 6 months prior to the PSC diagnosis, was significantly lower in AAs (41%) compared to NHWs (67%) (*p*=0.001). The baseline median AOM score was greater in AA compared to NHWs and HWs (*p*=0.001) but no differences in MRS (*p*=0.98) or MELD (*p*=0.25) were observed. The frequency of cirrhosis at baseline was lower in HWs (12.0%) compared to NHWs (12.0% vs. 27.2%, *p*=0.02) but did not differ between NHWs and AAs (*p*=0.9).

**TABLE 1:** Key clinical characteristics

	Non-Hispanic White (n=661)	African American (n=85)	Hispanic White (n=50)	*p* all groups	*p* NHW vs AA	*p* NHW vs HW
Age at enrollment, years	48.0 (35.0–61.0)	46.0 (37.0–56.0)	43.0 (35.0–56.0)	0.53	0.50	0.34
Age at imaging/histology diagnosis^a^, y	39.0 (26.0–53.0)	40.0 (27.0–49.0)	39.0 (30.0–49.0)	0.86	0.61	0.86
Age at biochemical diagnosis^b^, y	37.0 (24.0–51.0)	35.5 (26.0–48.0)	37.0 (30.0–46.0)	0.89	0.67	0.87
Sex, male, n (%)	430 (65.2)	47 (55.3)	28 (56.0)	0.11	0.07	0.19
PSC type, n (%)	—	—	—	0.21^c^	0.26	0.18
Large duct	551 (83.4)	73 (85.9)	39 (78.0)	—	—	—
Small duct	64 (9.7)	4 (4.7)	4 (8.0)	—	—	—
PSC/AIH overlap	46 (7.0)	8 (9.4)	7 (14.0)	—	—	—
IBD, n (%)	501 (75.8)	44 (51.8)	34 (68.0)	<0.0001	<0.0001	0.22
IBD type, n (%)	—	—	—	0.82^c^	0.60	0.81
Ulcerative colitis	373 (74.5)	35 (79.5)	27 (79.4)	—	—	—
Crohn disease	111 (22.2)	7 (15.9)	6 (17.7)	—	—	—
Indeterminate colitis	17 (3.4)	2 (4.5)	1 (2.9)	—	—	—
Cirrhosis at baseline^d^, n (%)	183 (27.7)	23 (27.1)	6 (12.0)	0.05	0.90	0.02
UDCA use (current or past), n (%)	434 (65.7)	60 (70.6)	39 (78.0)	0.08	0.03	0.62
Hemoglobin, g/dL	13.5 (12.2–14.9)	11.6 (10.8–12.9)	13.3 (11.6–14.4)	<0.0001	<0.0001	0.14
Platelets, ×10^6^	215.0 (148.0–286.0)	255.0 (187.0–354.0)	249.0 (209.0–313.0)	0.003	0.02	0.01
Albumin, mg/dL	4.0 (3.5–4.3)	3.7 (3.1–4.1)	4.0 (3.5–4.4)	0.004	0.002	0.55
Alkaline phosphatase, IU	201.0 (121.0–352.0)	233.0 (144.0–446.0)	185.5 (105.0–349.0)	0.17	0.08	0.60
Aspartate aminotransferase, IU	46.0 (30.0–83.0)	57.0 (40.0–113.0)	37.5 (26.5–94.5)	0.07	0.04	0.36
Alanine aminotransferase, IU	53.0 (30.0–96.0)	57.0 (37.0–113.0)	38.5 (28.5–108.5)	0.44	0.36	0.42
Total bilirubin, mg/dL	0.9 (0.6–2.0)	0.8 (0.5–1.9)	0.8 (0.5–2.1)	0.40	0.50	0.21
International normalized ratio	1.1 (1.0–1.2)	1.0 (1.0–1.3)	1.0 (0.9–1.1)	0.007	0.92	0.002
MELD Score	9.4 (9.4–11.1)	9.4 (9.4–11.4)	9.4 (9.4–9.5)	0.45	0.67	0.25
Mayo Risk Score	0.03 (−0.8 to 1.1)	0.02 (−0.7 to 1.0)	0.1 (-0.5–0.5)	0.98	0.83	0.94
Amsterdam-Oxford Model	14.3 (13.4–15.2)	15.1 (14.1–15.7)	14.5 (14.2–15.4)	0.001	0.002	0.03

a First documented abnormal cholangiogram for large-duct PSC or liver biopsy for small-duct PSC.

b First documented abnormal liver biochemistry.

c Fisher exact test. Quantitative variables are expressed as the median (interquartile range). Categorical variables are expressed as an absolute number (percentage).

d Cirrhosis defined by cirrhotic morphology on imagine, transient elastography >14.4 kPa, MRE >4.93 kPa, APRI >2, or reported cirrhosis with stage 4.

Abbreviation: MRE, Magnetic resonance elastography.

### Delay in diagnosis

Median (IQR) disease duration from the first abnormal cholangiogram or liver biopsy was 69.0 (20.0–120.5), 64 (29.0–135.0), and 64.0 (26.5–118.5) months in NHWs, AAs, and HWs, respectively; this did not differ by race or ethnicity (*p*=0.77). Given that delays in diagnosis are common in PSC, particularly in early asymptomatic stages when abnormal liver biochemistries may be the only indication, we sought to determine if variability in delay in diagnosis introduced bias. For this analysis, we defined diagnostic delay as the time (in months) between the first recorded abnormal liver biochemistry and the first abnormal cholangiogram or liver histology. A total of 524 NHW, AA, and HW patients had dates recorded for the first abnormal biochemistry and the first abnormal cholangiogram or liver biopsy. There was no difference in the median diagnostic delay [NHWs 4.0 (IQR 0.0–36.0), AAs 0.0 (IQR 0.0–25.0), HWs 5.0 (IQR 0.0–36.0) months, *p*=0.79]. Although diagnostic delays of >12 months were common, occurring in 206 (39.3%) of the 524 patients, frequency did not differ significantly between NHWs (167/423, 39.5%), AAs (21/64, 32.8%), or HWs (18/37, 48.6%) (*p*=0.37; Table 2).

**TABLE 2:** Time between first abnormal liver biochemistry and first abnormal cholangiogram or liver histology in months (n*=*524)

Time (mo)	Non-Hispanic White (n*=*423), n (%)	Black or African American (n*=*64), n (%)	Hispanic White (n*=*37), n (%)	*p*
<12	256 (60.5)	43 (63.2)	19 (51.4)	0.37^a^
12–24	40 (9.5)	3 (4.7)	5 (13.5)	—
24–36	21 (5.0)	6 (9.4)	3 (8.1)	—
≥36	106 (25.0)	12 (18.7)	10 (27.0)	—

a Fisher exact test.

### Outcomes

Among all 850 patients, the median (IQR) follow-up was 94 (39–147) months, during which 118 (13.8%) patients had a liver transplant and 40 (4.7%) died. Among NHW, AA, and HW patients, the median follow-up was 94 months. A total of 112 (14.1%) patients had a liver transplant during follow-up, and 38 (4.8%) died (Table 3). An HD event (ascites, variceal bleed, or HE) occurred in 137 patients (16.1%); hepatobiliary malignancy was diagnosed in 36 (4.2%). Colorectal neoplasia was diagnosed in 36 patients (4.2%).

**TABLE 3:** Causes of death, hepatic decompensation, and IBD-related events by race/ethnicity in 850 patients with PSC

	NHW (n=661)	AA (n=85)	HW (n=50)	Other/unknown (n=54)
Transplant	92 (13.9)	16 (18.8)	4 (8.0)	6 (11.1)
Death (cause)				
Liver failure	10 (1.5)	2 (2.4)	1 (2.0)	1 (1.9)
Sepsis (other infection)	2 (0.3)	1 (1.2)	0	0
Hepatobiliary cancer	3 (0.5)	2 (2.4)	0	0
Colon cancer	1 (0.2)	0	0	0
Other cancer	3 (0.5)	0	0	0
Other/unknown	9 (1.4)	2 (2.4)	0	1 (1.9)
Liver transplant complications	2 (0.3)	0	0	0
HD events (first event)				
Ascites	88 (13.3)	12 (14.1)	2 (4.0)	3 (5.6)
Variceal bleed	29 (4.4)	4 (4.7)	2 (4.0)	2 (3.7)
HE	54 (8.2)	9 (10.6)	2 (4.0)	2 (3.7)
Colonic neoplasia				
Dysplasia (high-grade)	9 (1.4)	0	1 (2.0)	2 (3.7)
Dysplasia (low-grade)	11 (1.7)	0	0	0
Indefinite for dysplasia	9 (1.4)	0	0	0
Adenocarcinoma	2 (0.3)	0	0	0
Colectomy	109 (16.5)	11 (12.9)	7 (14.0)	6 (11.1)

Abbreviations: AA, African American; HD, hepatic decompensation; HW, Hispanic White; IBD, inflammatory bowel disease; NHW, non-Hispanic White; PSC, primary sclerosing cholangitis.

**FIGURE 1:**
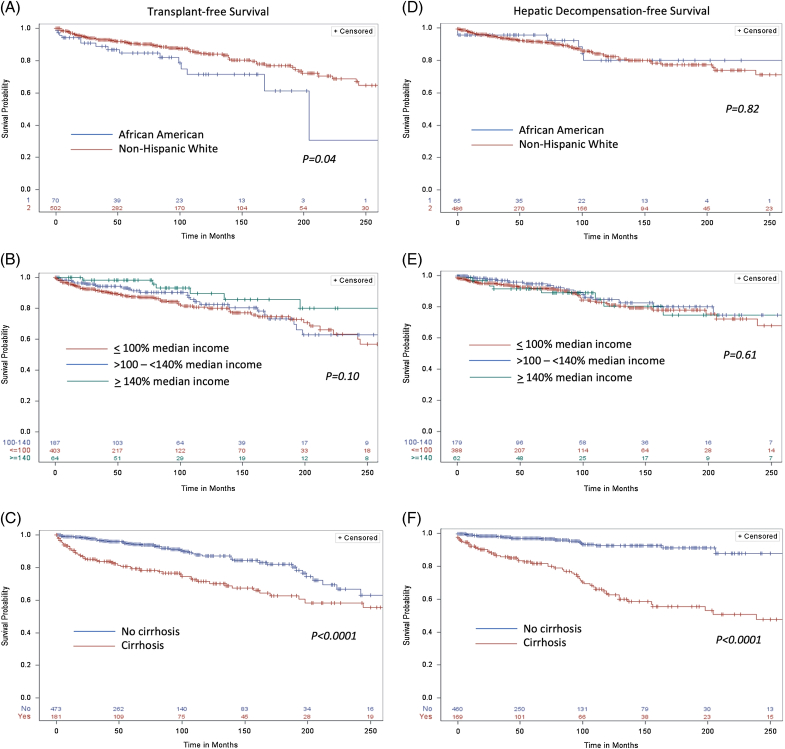
Transplant-free survival (A–C) and hepatic decompensation–free survival (D–F) from the time of first abnormal cholangiogram or liver histology by race (A and D), socioeconomic status (B and E), and presence of cirrhosis (C and F).

To account for the potential effects of delay in diagnosis of PSC, we performed a sensitivity analysis to evaluate time to clinical events using a start date from the first abnormal cholangiogram/histology or the first abnormal liver biochemistry. Clinical events were grouped as either death or liver transplantation or as any HD event. From the time of the first abnormal cholangiogram/histology, HD-free survival did not differ between AAs and NHWs (*p*=0.82), but TFS was significantly worse in AA compared to NHWs (*p*=0.04; Figure 1). Similar results were found when the start time of the first abnormal liver biochemistry was used (Supplemental Figure S1, http://links.lww.com/HC9/A761), the analysis was restricted to those with IBD (Supplemental Figure S2, http://links.lww.com/HC9/A761), or when deaths were limited to liver-related or sepsis (Supplemental Figure S3, http://links.lww.com/HC9/A762).

Those with a household income below the statewide median did not differ in HD-free survival time compared to those above the median, but they had significantly lower TFS from the first abnormal liver biochemistry (*p*=0.04) with a trend toward lower TFS from the first abnormal cholangiogram/histology (*p*=0.10; Figure 1 and Supplemental Figure S1, http://links.lww.com/HC9/A761). Patients with cirrhosis had significantly worse HD-free survival and TFS compared to those without cirrhosis, regardless of the start time considered (*p*<0.0001).

### Impact of race/ethnicity and SES on outcomes of interest

We examined several factors previously associated with death or liver transplant: age at diagnosis, sex, PSC type, IBD type, international normalizes ratio; platelet counts, and serum levels of alkaline phosphatase, total bilirubin, albumin, alanine aminotransferase, and AST. We also included race/ethnicity, SES, and cirrhosis in the analysis. Significant independent predictors of death or liver transplant included age at diagnosis (1.04, 1.02–1.06, *p*<0.0001), total bilirubin (1.06, 1.04–1.08, *p*<0.0001), and albumin (0.44, 0.33–0.61, *p*<0.0001; Table 4). AA race was a significant predictor of TFS in univariate analysis but did not reach significance in the multivariable analysis. Age at diagnosis, total bilirubin, and albumin were also significant predictors of HD (Supplemental Table S3, http://links.lww.com/HC9/A760). AA race was not a significant predictor of HD, but cirrhosis—which was not a predictor of death or liver transplant—had a strong effect on HD. A similar analysis excluding race/ethnicity did not meaningfully change the results (Table 4).

**TABLE 4:** Multivariate Cox proportional hazard analysis with and without race/ethnicity as a variable for death or liver transplant in the CALiD cohort (n*=*850)

	With race/ethnicity		Without race/ethnicity	
Parameter	HR (95% CI)	*p*	HR (95% CI)	*p*
Age at diagnosis^a^	1.04 (1.03–1.06)	<0.0001	1.04 (1.02–1.06)	<0.0001
Race/ethnicity (reference NWH)				
Black or African American	1.88 (0.88–4.00)	0.10	—	—
Hispanic White	0.58 (0.14–2.45)	0.46	—	—
Other/unknown	0.97 (0.34–2.73)	0.95	—	—
Cirrhosis	1.03 (0.57–1.87)	0.92	1.05 (0.58 – 1.90)	0.87
Total bilirubin	1.07 (1.04–1.09)	<0.0001	1.07 (1.05–1.09)	<0.0001
Albumin	0.45 (0.33–0.61)	<0.0001	0.45 (0.33–0.61)	<0.0001
ALT	1.00 (1.00–1.00)	0.25	1.00 (1.00–1.00)	0.35
AST	1.00 (1.00–1.00)	0.84	1.00 (1.00–1.00)	0.64
ALP	1.00 (1.00–1.00)	0.91	1.00 (1.00–1.00)	0.82
INR	1.46 (1.00–2.16)	0.06	1.43 (1.00–2.10)	0.07
Platelets	1.00 (1.00–1.00)	0.30	1.00 (1.00–1.00)	0.28
SES status^b^ (reference <100)				
100–140	0.97 (0.57–1.65)	0.91	0.95 (0.57–1.61)	0.86
>140	0.80 (0.33–1.94)	0.62	0.81 (0.33–1.96)	0.64

*Note:* Adjusted for sex primary sclerosing cholangitis type, and inflammatory bowel disease.

a Based on the first abnormal liver biochemistry.

b % statewide median household income based on residence Zip code.

Abbreviations: ALT, alanine aminotransferase; ALP, alkaline phosphatase; AST, aspartate aminotransferase; CALiD, Consortium for Autoimmune Liver Disease; INR, international normalizes ratio; NHW, non-Hispanic White; SES, socioeconomic status.

### Performance of predictive models in AA with PSC

**FIGURE 2:**
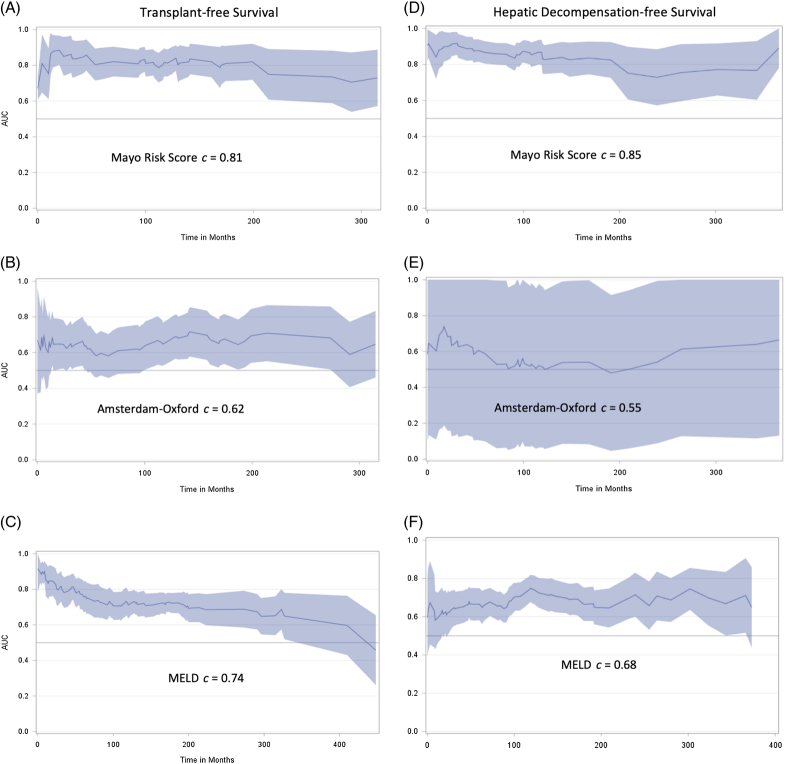
Performance of Mayo Risk Score (A and D), Amsterdam-Oxford Score (B and E), and MELD score (C and F) to predict transplant-free survival (A–C) and hepatic decompensation–free survival (D–F) in the total Consortium for Autoimmune Liver Disease cohort (n=850). Graphs represent Harrell c-index (line) with a 95% CI (shaded area) over time from diagnosis set at the first abnormal cholangiogram or liver biopsy. Abbreviation: MELD, Model for End-stage Liver Disease.

To determine whether race influenced prognostic models of PSC, we added AA race to the MRS, AOM, and MELD scores. We found no significant effect on proportional hazard analysis (Table 5). We also compared the performance of the models in all 850 patients of the cohort (Figure 2) and if the addition of AA race improved the model performance at 1, 5, and 10 years (Table 5). Overall, MRS and MELD performed well, with Harrell c-statistic of 0.81 (0.73–0.88) and 0.74 (0.66–0.77) for TFS, respectively, with the AOM performing less well with a c-statistic of 0.62 (0.55–0.70). The inclusion of AA race increased the c-statistic at 1 year for MRS and AOM from 0.64 (0.16–1.00) to 0.81 (0.64–1.00) and 0.54 (0.38–0.74) to 0.71 (0.55–0.89), respectively, but not at 5 and 10 years. Notably, although MELD performed consistently at 1, 5, and 10 years, MRS and AOM improved at 5 and 10 years. To test for miscalibration, we preformed the Greenwood-Nam-D’Agostino goodness-of-fit test on all 3 models with and without race. In all cases, the *p*-value was >0.05, indicating no evidence of miscalibration.

**TABLE 5:** Effect of African American race on prognostic models of transplant-free survival in PSC

Proportional hazard analysis^a^			Harrell c-index (95% CI)				GND test
Parameter	HR (CI 95%)	*p*	Model	1 y	5 y	10 y	*p* (χ^2^)
MRS	1.89 (1.58–2.26)	<0.0001	MRS	0.64 (0.16–1.00)	0.83 (0.72–0.93)	0.82 (0.72–0.91)	0.09 (6.6)
AA race	0.44 (0.13–1.53)	0.20	MRS + AA	0.81 (0.64–1.00)	0.85 (0.76–0.95)	0.83 (0.73–0.94)	0.09 (7.9)
AOM	1.33 (1.17–1.52)	<0.0001	AOM	0.54 (0.38–0.74)	0.59 (0.49–0.69)	0.60 (0.50–0.69)	0.25 (2.8)
AA race	0.84 (0.39–1.85)	0.67	AOM + AA	0.71 (0.55–0.89)	0.65 (0.55–0.75)	0.63 (0.53–0.72)	0.56 (2.1)
MELD	1.10 (1.08–1.13)	<0.0001	MELD	0.73 (0.55–0.87)	0.78 (0.70–0.85)	0.74 (0.67–0.80)	0.63 (3.4)
AA race	1.00 (0.54–1.85)	0.99	MELD + AA	0.73 (0.62–0.84)	0.72 (0.53–0.75)	0.69 (0.60–0.77)	0.91 (1.0)

a Multivariable model.

Abbreviations: AA, African American; AOM, Amsterdam-Oxford model; GND, Greenwood-Nam-D’Agostino Calibration Test; MELD, Model for End-stage Liver Disease; MRS, Mayo risk score.

## DISCUSSION

In a large, racially diverse study of patients with PSC in North America, we observed that AA patients as a group have a reduced TFS compared to NHWs, but after adjusting for clinical factors, race was not a significant predictor of TFS or HD-free survival. Utilizing data from the United Network for Organ Sharing (UNOS), we previously found that AA with PSC was listed for a liver transplant at younger ages and with higher MELD scores compared to NHWs.^16^ In a recent single-center study, AAs were also noted to be diagnosed at a younger age and with more advanced disease.^20^ Analysis of administrative data from the United Kingdom also reported that African Caribbean heritage was associated with a younger age at diagnosis and a greater risk for liver transplantation or PSC-related death.^9^ Although the UK study included a large cohort, it included only patients with concomitant IBD, a limitation that could exclude nearly half of the African Caribbean population with the PSC. Importantly, due to the lack of detailed clinical data, including liver biochemistries, these prior analyses could not fully determine if the effects of race were independent of clinical factors such as total bilirubin and albumin, which have been the strongest predictors of clinical outcomes in prior studies.^11^,^13^,^14^ In contrast, our current study was inclusive of all patients with PSC at 16 expert centers, with detailed clinical data and outcomes. Thus, our findings of a lower TFS in AA compared to NHWs on Kaplan-Meyer analysis and on univariate time-to-event analysis are consistent with prior studies.^20^,^21^ However, after adjustment for clinical factors that have previously been found to be associated with TFS and HD from PSC, race was no longer significantly associated with TFS.

The lack of an effect of race on TFS is rather surprising given the disparities minority populations experience in liver transplantation. Compared to NHWs, AA are listed for liver transplant less frequently relative to death from end-stage liver disease^22^ and to the overall population.^23^ When listed, AAs are listed with higher MELD scores^24^ and have lower rates of living donor liver transplantation compared to NHWs, specifically for cholestatic liver diseases.^25^ AAs listed for liver transplantation for autoimmune liver diseases also have greater waitlist mortality.^26^

Health disparities, in general, may arise from social, economic, and geographic differences, as well as from biological differences. A prior study of 449 patients with PSC, including 45 AA patients, found a significant interaction between community socioeconomic factors and race which reduced the effects of race on survival.^20^ In our current study, we found no significant impact of SES on either TFS or HD-free survival. Notably, the time of our study included the expansion of Medicaid under the Affordable Care Act, which has increased access to medical care, including listing for liver transplantation,^27^ and has been associated with a significant decrease in liver-related mortality.^28^ Thus, some of the negative effects of lower SES previously observed may have been mitigated in recent years, including during the period of our study.

Delayed diagnosis, leading to potential bias in the estimation of disease progression rates, was not apparent in our study. Although we have shown that the proportion of AAs in PSC cohorts is similar to that of the population from which they are recruited,^15^ PSC has often been characterized as a disease among NHWs, leading us to speculate that lack of awareness of PSC in AAs could cause delays in recognition and diagnosis. However, we found no difference between NHWs and AA in time from the first abnormal liver biochemistry to the diagnostic cholangiogram or liver biopsy. In addition, we found no differences in age at diagnosis or measures of disease severity at baseline, including cirrhosis, total bilirubin, or MELD score. Further, race did not affect time to HD, even on univariate analysis. In the absence of an effective medical therapy that alters the natural course of PSC, these findings strongly support a disease progression in AA, which is similar to NHWs.

Despite a comparable progression of PSC-related liver disease, this study confirmed our earlier observation that AA patients with PSC have a lower rate of IBD than NHWs.^16^ In our prior study of waitlisted patients with PSC, we could not exclude the possibility that the lower rate of IBD was due to a lower rate of colonoscopy and thus missed diagnoses of IBD. The rate of colonoscopy in our current study, including patients not listed for liver transplantation, was similar between AAs and NHWs, supporting a true difference in IBD frequency. Prior studies have associated Crohn disease with better clinical outcomes compared to those with ulcerative colitis or no IBD,^6^,^29^ but we were unable to validate this finding in our cohort. Among those patients with IBD, there was no difference in the distribution of IBD type between NHWs and AAs.

Another important finding of our study was the validation of prognostic models of TFS in PSC which were developed and validated in cohorts with limited diversity. The incorporation of race did improve the performance of the MRS and AOM survival models at 1 year, but not at 5 and 10 years. Both models use AST rather than alanine aminotransferase and, in our cohort, AST was significantly higher in AA compared to NHW patients potentially leading to an overestimation of risk. Regardless, our findings further validate the use of these models in racially and ethnically diverse populations for risk stratification beyond 1 year.

Our study had many strengths, including a large, racially, and ethnically diverse cohort of patients with PSC and detailed clinical data available for analysis. In addition, we limited our data collection to reflect a contemporary patient cohort. Further, we used HD as an outcome to confirm our findings of TFS. Given that geography, organ allocation paradigm, and donor type all impact the timing of liver transplant, TFS may not be the best clinical outcome for use in real-world studies or clinical trials of PSC. In contrast, because there are currently no effective treatments for PSC, HD is a clinical end point less subject to the impact of nonbiological variables and potentially a better outcome for research purposes. There are, however, some limitations to acknowledge. Although well represented in the CALiD registry, the cohort of AA patients with PSC was still relatively small. The inability to demonstrate an effect of AA race may have been due to the limitations of the sample size of AA in the cohort. However, the effect size from the UK study and the narrow confidence intervals we observed suggest that the effect of race on TFS and/or HD-free survival would be small if it does exist. In addition, we did not see a protective effect of small-duct PSC as previously reported, likely due to the small number of patients with small-duct PSC in our cohort, and there may be inherent biases because all patients were recruited at large academic medical centers with expertise in PSC. Therefore, our results may not be generalizable to other health systems.

In conclusion, AA patients with PSC have a lower frequency of IBD, but our results suggest that the natural history of PSC, including progression to liver transplantation/death and HD, is similar in AAs and NHWs. Current prognostic models perform well in a diverse population of patients with PSC. Future real-world studies and clinical trials of PSC must include racially and ethnically diverse populations to ensure that treatment effects are equal across diverse groups of patients.

## Supplementary Material

SUPPLEMENTARY MATERIAL
