# Supplementary material for: African American race does not confer an increased risk of clinical events in patients with primary sclerosing cholangitis
Source: Hepatol Commun. 2024 Jan 29;8(2):e0366. doi: 10.1097/HC9.0000000000000366 (PMC10830082; doi:10.1097/HC9.0000000000000366)
Supplement: SUPPLEMENTARY MATERIAL [file hc9-8-e0366-s001.docx]

**SUPPLEMENTAL MATERIAL**

**Table S1.** Patient Enrollment by Center (number, %)

| **Center** | **Non-Hispanic White**  (n=661) | **Black or African American**  (n=85) | **Hispanic-White**  (n=50) | **Other/Unknown**  (n=54) |
| --- | --- | --- | --- | --- |
| BWH | 10 (1.5%) | 0 | 0 | 2 (3.7%) |
| Henry Ford | 24 (3.6%) | 11 (12.9%) | 0 | 0 |
| Indiana University | 105 (15.9%) | 10 (11.8%) | 1 (2.0%) | 3 (5.6%) |
| Kaiser Permanente Northwest | 28 (4.2%) | 0 | 1 (2.0%) | 2 (3.7%) |
| MGH | 83 (12.6%) | 4 (4.7%) | 0 | 7 (13.0%) |
| Mayo Clinic Arizona | 28 (4.2%) | 3 (3.5%) | 2 (4.0%) | 1 (1.9%) |
| OHSU | 27 (4.1%) | 0 | 0 | 5 (9.3%) |
| Tufts Medical Center | 8 (1.2%) | 0 | 0 | 3 (5.6%) |
| University of Miami | 65 (9.8%) | 28 (32.9%) | 34 (68.0%) | 6 (11.1%) |
| UC Davis | 68 (10.3%) | 10 (11.8%) | 5 (10.0%) | 6 (11.1%) |
| UCSF | 3 (0.5%) | 1 (1.2%) | 0 | 0 |
| University of Colorado | 53 (8.0%) | 3 (3.5%) | 4 (8.0%) | 3 (5.6%) |
| UPenn | 74 (11.2%) | 10 (11.8%) | 1 (2.0%) | 8 (14.8%) |
| University of Alberta | 41 (6.2%) | 0 | 0 | 6 (11.1%) |
| University of Utah | 3 (0.5%) | 0 | 0 | 0 |
| University of Washington | 0 | 0 | 0 | 1 (1.9%) |
| Yale | 41 (6.2%) | 5 (5.9%) | 2 (4.0%) | 1 (1.9%) |

**Table S2.** Frequency of criteria used for the diagnosis of cirrhosis at baseline (n = 850)

| **Criteria** | **Meeting Criteria (n, %)** |
| --- | --- |
| Any | 226 (26.6%) |
| Cirrhotic Morphology from 1^st^ MRI | 75 (8.8%) |
| Cirrhotic Morphology from 1^st^ Ultrasound | 47 (5.5%) |
| Cirrhotic Morphology from 1^st^ CT | 68 (8.0%) |
| TE > 14.4 kPa | 43 (5.1%) |
| MRE > 4.93 kPa | 22 (2.6%) |
| APRI > 2 | 98 (11.5%) |
| Biopsy stage 4 | 22 (2.6%) |

**Table S3.** Additional Clinical Characteristics

|  | Non-Hispanic White  (*n = 661*) | African American  (*n = 85*) | Hispanic White  (*n = 50*) | P-Value  *All groups* | P-Value  *NHW vs AA* | P-Value  *NHW vs HW* |
| --- | --- | --- | --- | --- | --- | --- |
| PSC Distribution |  |  |  | 0.59 | 0.65 | 0.41 |
| Intrahepatic only | 115 (48.7%) | 12 (41.4%) | 9 (60%) |  |  |  |
| Intrahepatic and extrahepatic | 99 (42.0%) | 13 (44.8%) | 6 (40%) |  |  |  |
| Extrahepatic only | 22 (9.3%) | 4 (13.8%) | – |  |  |  |
| Cirrhotic Morphology from 1^st^ MRI | 60 (9.1%) | 9 (10.6%) | – | 0.008 | 0.02 | 0.03 |
| Cirrhotic Morphology from 1^st^ US | 39 (5.9%) | 4 (4.7%) | 1 (2.0%) | 0.11 | 0.28 | 0.10 |
| Cirrhotic Morphology on 1^st^ CT | 56 (8.5%) | 7 (8.2%) | 1 (2.0%) | 0.04 | 0.23 | 0.02 |
| TE > 14.4 kPa | 32 (4.8%) | 7 (8.2%) | 1 (2.0%) | 0.24 | 0.19 | 0.36 |
| APRI > 2 | 82 (12.4%) | 10 (11.8%) | 3 (6.0%) | 0.40 | 0.87 | 0.18 |
| Stage 4 on biopsy | 14 (2.1%) | – | 2 (4.0%) | 0.25 | 0.18 | 0.39 |
| Sodium, *mg/dL* | 139.0  (137.0–141.0) | 138.0  (136.0–140.0) | 139.0  (137.0–141.0) | 0.08 | 0.03 | 0.75 |
| Creatinine, *mg/dL* | 0.8  (0.7–1.0) | 0.8  (0.7–1.0) | 0.8  (0.7–1.0) | 0.66 | 0.97 | 0.37 |

Quantitative variables are expressed as the median (interquartile range). Categorical variables are expressed as an absolute number (percentage).

**Table S4.** Univariate and multivariate Cox proportional hazard analysis for hepatic decompensation in the CALiD cohort (n = 850)

|  |  | **Univariate** | | **Multivariate** | |
| --- | --- | --- | --- | --- | --- |
| **Parameter** |  | **HR (CI 95%)** | **P-Value** | **HR (CI 95%)** | **P-Value** |
| Age at Diagnosis^1^ |  | 1.05 (1.03–1.06) | <0.0001 | 1.04 (1.02–1.06) | 0.0001 |
| Gender |  | 0.89 (0.59–1.36) | 0.59 | – | – |
| Race/Ethnicity (*reference NWH*)  Black or African American  Hispanic White  Other/Unknown |  | 1.09 (0.56–2.10)  0.40 (0.10–1.64)  0.94 (0.38–2.32) | 0.81  0.20  0.89 | 1.34 (0.47–3.84)  0.76 (0.18–3.17)  1.70 (0.60–4.79) | 0.58  0.70  0.32 |
| Cirrhosis |  | 4.32 (2.84–6.56) | <0.0001 | 4.22 (2.00–8.90) | 0.0002 |
| Total Bilirubin |  | 1.10 (1.07–1.13) | <0.0001 | 1.06 (1.03–1.10) | 0.001 |
| Albumin |  | 0.38 (0.29–0.49) | <0.0001 | 0.66 (0.46–0.95) | 0.02 |
| ALT |  | 1.00 (1.00–1.00) | 0.63 | – | – |
| AST |  | 1.00 (1.00–1.00) | 0.11 | – | – |
| ALP |  | 1.00 (1.00–1.00) | 0.52 | – | – |
| INR |  | 2.03 (1.42–2.90) | <0.0001 | 1.00 (0.57–1.62) | 0.88 |
| Platelets |  | 1.00 (1.00–1.00) | <0.0001 | 1.00 (1.00–1.00) | 0.28 |
| PSC subtype (*Large duct as reference*) |  |  |  |  |  |
| PSC/AIH Overlap |  | 1.15 (0.55–2.38) | 0.72 | – | – |
| Small Duct PSC |  | 0.85 (0.43–1.69) | 0.64 | – | – |
| IBD type (*Crohn’s vs other*) |  | 0.74 (0.38–1.43) | 0.37 | – | – |
| SES Status^2^ (*Reference < 100*)  100–140  >140 |  | 0.84 (0.53–1.33)  0.83 (0.45–1.55) | 0.45  0.56 | –  – | –  – |

^1^Based upon first abnormal liver biochemistry ^2^% statewide median household income based upon residence Zip code.
