# Supplementary material for: African American race does not confer an increased risk of clinical events in patients with primary sclerosing cholangitis
Source: Hepatol Commun. 2024 Jan 29;8(2):e0366. doi: 10.1097/HC9.0000000000000366 (PMC10830082; doi:10.1097/HC9.0000000000000366)
Supplement: SUPPLEMENTARY MATERIAL [file hc9-8-e0366-s002.docx]

**Supplemental Figure S1.** Transplant-free survival (A-C) and hepatic decompensation-free survival (D-F) from time of first abnormal liver biochemistry by race (A and D), socioeconomic status (B and E), and cirrhosis (C and F).


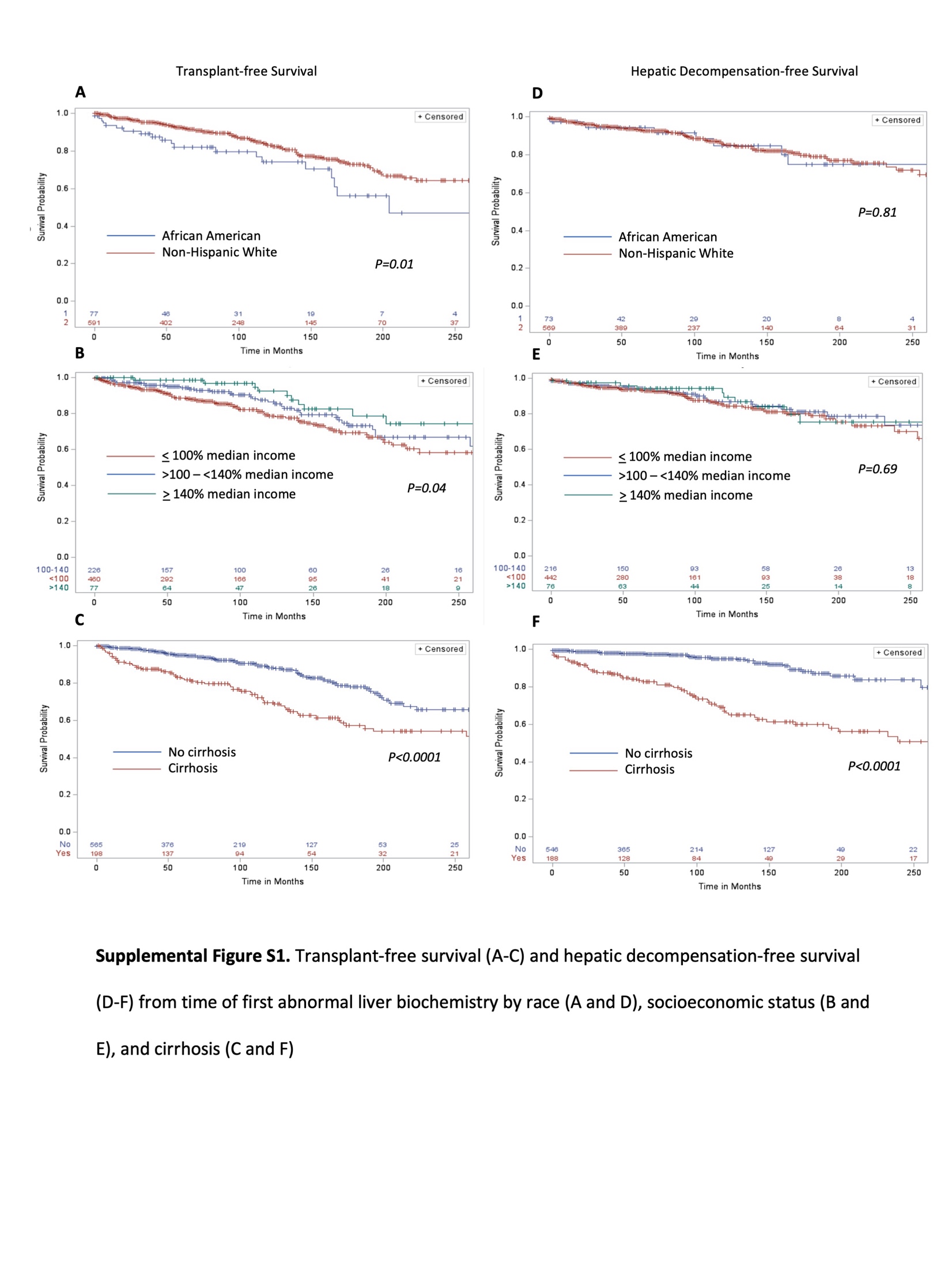


**Supplemental Figure S2.** Transplant-free survival (A and B) and hepatic decompensation-free survival (C and D) from time of first abnormal liver biochemistry (A and C) and from first abnormal cholangiogram or liver histology (B and D) by race among patients with inflammatory bowel disease (IBD).

**
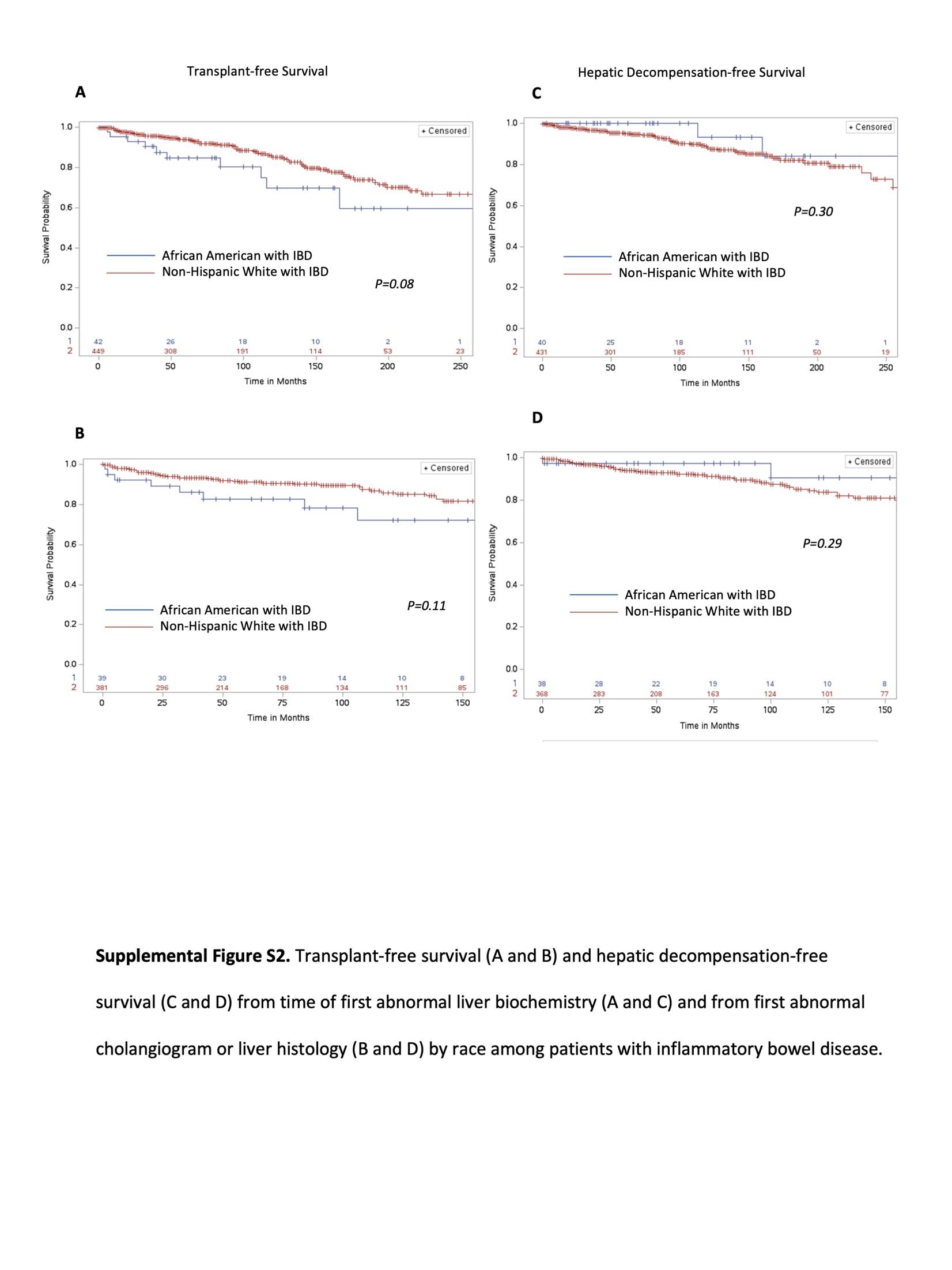
**
