# Supplementary figures and images for: African American race does not confer an increased risk of clinical events in patients with primary sclerosing cholangitis
Source: Hepatol Commun. 2024 Jan 29;8(2):e0366. doi: 10.1097/HC9.0000000000000366 (PMC10830082; doi:10.1097/HC9.0000000000000366)

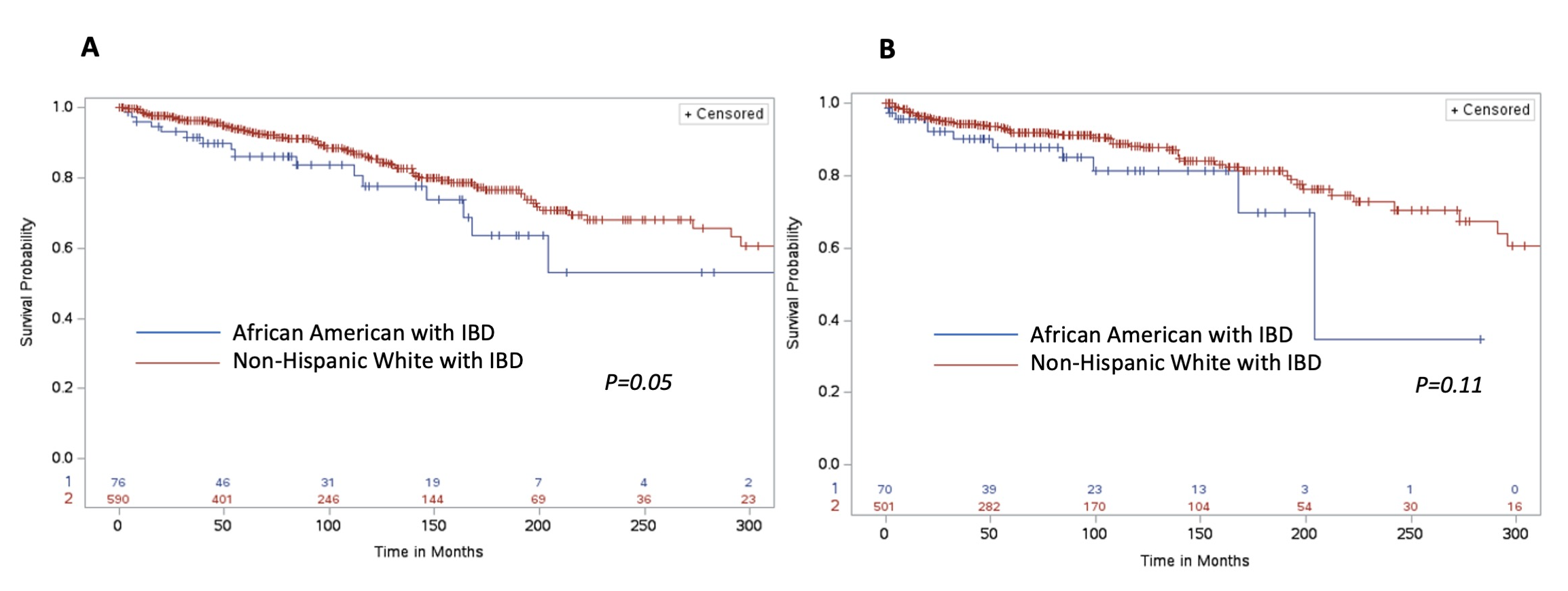

Supplement: SUPPLEMENTARY MATERIAL [file hc9-8-e0366-s003.tiff]
